# American Academy of Dental Science

**Published:** 1896-08

**Authors:** 

**Affiliations:** American Academy Dental Science


### Reports of Society Meetings.

### AMERICAN ACADEMY OF DENTAL SCIENCE.

THE regular monthly meeting of the American Academy of Dental Science was held at Young's Hotel, Wednesday evening, March 4, at six o'clock, President Andrews in the chair.

*President Andrews.*—Gentlemen, the Academy is extremely fortunate this evening in having as our essayist one who I am sure you will all be very glad to hear. It gives me great pleasure to introduce to you Frederic C. Cobb, M.D., of Boston, whose subject is "Empyema of the Antrum of Highmore," with exhibition and illustration of cases by Voltolini's method.

(For Dr. Cobb's paper, see page 405.)

DISCUSSION.

*Dr. Cobb.*—This method of transillumination is nothing new. The only interest which I claim for it is the fact that it has not been publicly shown on any patients in Boston. It was gotten up by Voltolini a good many years ago, and like most new things was at first overpraised and then again undervalued, and more or less lost sight of. In the cases in which I have used it, in both hospital and private practice, it has shown in almost all instances very satisfactory results.

It is not to be accepted as a conclusive argument for the existence of antrum disease, but simply as one sign which, in combination with other symptoms, helps to make our diagnosis more accurate.

(The lights in the room were then turned out, and the essayist presented several subjects, placing in the mouth of each a tiny electric light, which transilluminated the face, the presence of disease being indicated by dark lines extending to the eye, sometimes on one side and in other cases on the opposite.)

*President Andrews.*—Gentlemen, this most interesting subject is before you. I am sure the essayist will be glad to answer any questions that any of the members may wish to ask, and I am in hopes the subject will be fully discussed by the members present.

*Dr. Wilson.*—The speaker referred to some old cases of antrum disease at the Massachusetts General Hospital, of which he said there was no record. I would like to ask about how old the records of those cases were?

*Dr. Cobb.*—The oldest case I could find mentioned was about twenty years ago. I did not mean to say that there was no record. What I meant was that the record was not so complete as I desired. I could not look up the patients themselves, who had probably wandered all over the country, so that I did not know how long their treatment lasted. If I could have talked with some of them, and learned how quickly their discharge ceased, that might have been valuable information. But in these hospital cases it is almost impossible to find the patients; they migrate all over the world and leave no address anywhere.

*Dr. Potter.*—Will Dr. Cobb please tell us what the distance is between the opening of the frontal sinus and the opening of the antrum?

*Dr. Cobb.*—The frontal sinus and the anterior ethmoidal and the antrum all drain into the infundibulum below the middle turbinate. If there is an obstruction to the outflow through the nares, the pus from any disease of the frontal or ethmoidal sinus is very likely to be forced into the antrum. If the middle turbinate is swollen,—and it often is swollen when there is a disease in any of these cavities,—the pus can run down into the antral opening and then down into the antrum. The distance between these openings varies. I should say that they average about one-eighth of an inch. The antrum could not easily force its secretions into the frontal sinus, but the frontal sinus can drain into the antrum.

*Dr. Smith.*—In the successful treating of these cases, is it always necessary to extract a tooth, providing a tooth is the offender? Or, to put the question in another way, suppose a putrescent pulp in a molar produced disease of the antrum, is it not possible to treat this disease through the roots of the molar? And when the antrum is cured fill the roots of the molar and preserve it in the mouth. Need further trouble in the antrum be expected from the tooth?

*Dr. Cobb.*—That opens up a very interesting question, and one that my experience does not enable me to decide. I was in hopes I might hear something touching upon that point from the gentlemen present this evening. In the case of the young man who came in this evening, it was very evident that the antral trouble was caused by a tooth, but the tooth was so far gone, and presented such enormous cavities, that it never occurred to me to consider whether it would be wise to do anything else than extract it. I think in all cases reported the procedure of curing any disease of the antrum is usually so long that most surgeons, if they can find a tooth which connects with the antrum, and which has given evidence of unsoundness, usually remove it and treat the antral disease through the tooth-socket. It seems to me that such a tooth might serve as a foreign body in the floor of an antrum, and that it would be wise to remove it. A good stream of water is, I think, imperative in the treatment of these cases.

*Dr. Pond.*—I would like to mention a case in my practice of antral disease with an opening externally on the face. It undoubtedly resulted from the death of a pulp in a bicuspid, and the tooth was kept because it preserved the opening. After a treatment of about two weeks the antrum healed, and there was no further discharge, and the root was filled, and there has never been any trouble from the case since. The case was treated more than a year ago and quite a large opening was made through the tooth.

*Dr. Cobb.*—I would like to ask in that case whether there was any discharge of pus through the nose?

*Dr. Pond.*—There did not seem to be. The discharge and the washing all came through the fistula in the face and the opening through the tooth.

*Dr. Cooke.*—I would like to ask Dr. Pond how he knows the fistula went into the antrum?

*Dr. Pond.*—You can easily pass a probe into the antrum.

*Dr. Ainsworth.*—I would report a case similar to that, only occurring in the first molar. It came into my hands perhaps six months ago. A probe readily entered the antrum through all three roots; there was quite an unusually large-sized opening through each apex and a fistulous opening through the gum draining the antrum. After a thorough cleansing and treatment at two sittings I filled the roots with gutta-percha, the crown with cement, and dismissed the case. Six weeks later the patient returned, when I found the parts perfectly healed.

*Dr. Werner.*—May I ask Dr. Cobb what antiseptic washes he uses?

*Dr. Cobb.*—At first I used mild solutions, as Seiler's tablets, Dobell's solution, and various other mild washes. Then I used peroxide of hydrogen, which I found rather irritating in most cases. I commenced at first with a solution of one in three, and then used it stronger, but in most of the cases it seems to irritate too much, even when I used weak solutions. After that I used iodoform in a solution of one in ten diluted with water, simply filling the antrum with it, and leaving it there for a little while, and then washing it out with alkaline solutions. Then I tried europhen, which did not show special merit. I have also tried a very mild corrosive, one in thirty thousand. I do not think it makes very much difference what we use in all these cases as far as the cure of the disease is concerned. I think there is some cause beyond all this that must be reached,—some foreign body, necroses of bone,—something that is not amenable to simply antiseptic washing. My experience with this trouble has been rather discouraging so far as the results from these antiseptic washes are concerned.

*Dr. Werner.*—Did you ever use glycothymoline?

*Dr. Cobb.*—Not in such cases.

*Dr. Ainsworth.*—I would like to ask Dr. Cobb if the operation of curetting the antrum is usually a painful one without the use of cocaine?

*Dr. Cobb.*—The only case in which I have had an opportunity to curette from the bottom up was in the case of one of the women here to-night, and in that case there seems to be no trouble anywhere but in the antrum. The curetting caused quite a little pain, but not so much but that she would be perfectly willing to have it done over again if I thought it would do any good. I put on quite a little pressure,—as much as I thought it was safe to use. She had, I am sure, some bare bone in the antrum.

*Dr. Ainsworth.*—I have in mind a case of curetting of a year and a half's duration. There seemed to be necrosed or bare bone over the bicuspids. I suspected possible trouble from a pulpless tooth. I could not, however, bring myself to believe that this was the cause, because the tooth had been well treated and had given no trouble after it was filled, and there was some distance between the apex of the root and the floor of the antrum, yet there was great sensitiveness when the curette was passed over that portion. Later this case cured readily after the burring out of the anterior ethmoid cells.

*Dr. Cobb.*—Did you see the case after it was burred out?

*Dr. Ainsworth.*—Not to make a critical examination of it. It seems to be permanently cured, however. I would add that in this case the patient became so despondent and discouraged at the slow progress made towards a cure that he was almost driven to suicide.

*Dr. Brackett.*—I have been very much impressed by the demonstrations that have been made before us here to-night, and I feel very grateful to the essayist for showing them. They seem to be very practical and helpful, and the subject has been placed before us in such a manner that we shall not readily forget what we have seen and heard.

It is impressive to think how much of pathology is demonstrated not only to be possible, but to be actually existing in this nasal region, in the group of tissues in the neighborhood which the essayist has mentioned in his paper, and the not remote region where adenoid growths flourish. It is impressive to think how much has been learned in recent years of all this comparatively new field of pathology, and also how much has been learned of the possible practical surgery and therapeutics for the very great benefit of patients who have been thus afflicted.

My experience with antral cases has given general corroboration to the statement of Dr. Garretson that a very material proportion of these cases appear to be dependent for their causation upon tooth-troubles. I have found a very large part of them to be dependent upon such tooth causation that to undertake the salvation of the tooth would be to incur the risk of imperfect treatment and consequent continuance of the antral disease. I have felt that the possibilities of harm to the patient in attempting to retain the tooth far out-balanced the possible future value of the tooth, and quite a share of the cases that I have seen yielded readily to removal of the causative influence in connection with the tooth. Some have not thus readily yielded and have required treatment for a long time.

I wish to speak of the fountain syringe at an elevation as a positively efficient means of flushing that I have used in one of these antral cases. In the treatment of this case Dr. Beverly Robinson, of New York, was with me. After the extraction of the molar tooth, which was the occasion of the antral trouble, we used this very thorough flushing, employing various forms of medication without getting full response until we came upon one which did seem to be followed by gratifying results,—namely, a teaspoonful of the tincture of iodine to the pint of tepid water. I think there is danger in these cases of over-medication; but if thorough washing out is indicated this is a means for its accomplishment.

*Dr. Eames.*—I am moved to speak from what has been said by the last speaker in regard to his cases generally proceeding from the teeth. My experience has been quite the reverse. In the first fifteen years of dental practice I never saw an antral case; but within the last six years, having had an opportunity to see patients who came to dispensaries, I have seen a number of them. Those that have been in my hands have, in the majority of cases, proceeded from the nose. I have read all authorities in regard to this disease, and I think Dr. Cobb will agree that those who believe that the cause most frequently proceeds from the teeth, and those who think that the origin of most cases can be found in the nose are equally divided. Those who have to deal with dental troubles think that the majority of antral troubles come from the teeth, while rhinologists claim that they come from the nose in the majority of cases.

In answer to the question raised by Dr. Smith, I can see how antral disease of short standing, brought about by the discharge of pus into the antrum from an abscessed tooth, might be cured by treatment through that tooth, which could then be filled and allowed to remain, but it strikes me that a majority of the cases could not be treated in that way. When we find so many antral cases that resist treatment for months and years, when there are one or more large openings into the antrum, we feel that such cases could not be successfully treated through the tooth alone.

I believe the ideal treatment is one in which two openings are practicable.

Referring again to the question of the origin of these antral troubles, I would like to cite one case which shows the possibility of error in diagnosing the teeth as the cause. The history of the case, briefly, is this: The symptoms were given by the patient, and a physician who saw the case diagnosed "ulcerated teeth," and lanced the gum. This not showing the desired effect, it finally resulted in the extraction of the teeth involved, followed by the ordinary treatment for antral trouble through the alveolar openings thus made. The case not getting well, was put into my hands, and on examination of the nose I found what seemed to be a polypus, and I took a fine wire and snared away several pieces of it. On looking at the pieces removed, they appeared to be more fibrous in character, not true polypus; and subjecting it to a microscopical examination, it was found to be a round-cell sarcoma, which extended from the nose down into the antrum, nearly filling it. After enlarging the opening made by the extracted teeth, I took my adenoid forceps, and was enabled to grasp masses of the growth, and remove it with very much less pain and less hemorrhage than by the ordinary process of curetting. These masses that came away would seem to show that the disease perhaps originated by an ordinary polypus in the nose.

*Dr. Smith.*—In neglected disease of the antrum will the essayist please tell us to what extent the disease involves contiguous tissue and to what complications follow?

*Dr. Cobb.*—I have seen cases which have lasted for many years. I think they are shown by fouler smell and more discharge; but that is about the only sign that I have noticed. I have not seen any very extensive necroses of the alveolar process resulting from long suppuration. My cases, as I say, have not been numerous enough, perhaps, to found any statement as to what the effect of long-continued neglect may be.

Simple empyema does not seem to extend beyond the limits of the antrum. Many of these cases report that they do not get much worse; they seem to keep along just about the same month after month.

*Dr. Taft.*—I would like to ask Dr. Cobb if there is not a possible correspondence between a chronic continuance of this disease and a chronic continuance of nasal catarrh?

*Dr. Cobb.*—No correspondence that I know of, excepting that they are serious as affecting the general health. Now, in the first one of these cases shown here to-night the woman weighed only one hundred and nineteen pounds when she came into the hospital for treatment. She was treated for empyema without any show of improvement at first, and it was a question whether she was progressively going down hill or not. Careful examination showed that there was no sign of malignancy in the case, and after a time she began to gain flesh and strength rapidly; she has now gained eleven pounds. So that keeping the antrum clear certainly has an effect upon the patient's general health.

*Dr. Smith.*—In regard to the treating of the antrum through the roots of teeth and saving the teeth, I have in mind one case where this treatment was not successful. My associate, Dr. Payne, asked my advice one day in regard to a case of antral disease caused by a first molar that was not only pulpless but thoroughly dead; and he wanted to know if it was not possible to cure the disease and save the tooth. I advised him to extract the tooth, but he decided to attempt the treatment of the antral disease through the tooth. He tried it a while, and gave it up; then he extracted the tooth. I have forgotten to ask him how much he treated it, or how persistent it was, and I will therefore ask him now how long it took to cure that case.

*Dr. Payne.*—The patient presented after having an opening drilled through the process to relieve an abscess that was forming. This drilling, by mistake, was carried to the antrum, and a syringeful of strong creosote was injected; but the effects of that were not apparent when the case came into my hands. She came to me with considerable trouble in this pulpless first molar, with a discharge of pus from the nose. The fistula, in the mean time, had closed. I removed the filling in the tooth, and found dressing in the roots, which I removed; enlarged the opening through the end of the palatal tooth, and washed out the antrum with a very weak solution of carbolic acid and water, perhaps one to two hundred and fifty or three hundred. The patient went away, and when I next saw her found that the pus had discharged through the palatal root and also through the nose. I continued this treatment through the root until the pus discharge had ceased. A dressing was put in the canals, and the cavity in the tooth stopped up, and the case went along for about four months with no trouble at all. She came in one day complaining of a severe pain in the affected region, and stating that she had had a severe cold, which caused a return of symptoms, and pus was again discharging through the nose. I removed the dressing, washed out the antrum, and found that the canals could not be stopped up without causing pain; and, as the patient so desired, I had the tooth extracted, washed out the sockets and antrum with a weak solution of carbolic acid, and it immediately healed.

*Dr. Smith.*—I would like to ask Dr. Cobb if he has used carbolic acid in any form for washing out the antrum?

*Dr. Cobb.*—I have not.

*Dr. Smith.*—I saw it recommended years ago for this purpose, and I still consider it a good remedy.

*Dr. Cobb.*—How strong do you use it?

*Dr. Smith.*—About one in two hundred and fifty to three hundred.

*Dr. Cobb.*—In washing the antrum we always have to consider the irritation of anything passing over the nasal mucous membrane. Dr. Payne spoke of the patient in his case having a cold, and thus starting up her antrum again. I have heard a good many patients complain of just that thing, but, if you will look into it, you will often find that it was not a cold which started up the antrum, but the antrum which caused the "stuffed up" feeling which the patient supposed to be a cold. If you cross-question them, you will find that their cold was simply one-sided, while the other side is absolutely clear, and the discharge is simply antral pus. That fact I did not discover for some little time, so that it is always advisable to find out whether a patient's definition of a cold is really a bilateral stoppage followed by simple mucous discharge, or a unilateral flow of offensive-smelling pus, sometimes not even preceded by any stopping.

*Dr. Banfield.*—When the disease was cured, you would expect these openings made into the antrum to close of themselves?

*Dr. Cobb.*—The artificial openings? Yes, they heal so quickly that one can hardly keep them open. Behind the vestibule in the nose the nasal secretion is very antiseptic, so they have the very best possible chance of healing.

*Dr. Taft.*—I would like to know how the essayist would diagnose a case of empyema,—that is, to distinguish it from a case of chronic nasal catarrh?

*Dr. Cobb.*—There are other forms of discharge that are offensive besides empyema. One is ozæna, which is accompanied by dry crusts and scabs, very shrunken turbinates, and dry-looking mucous membrane. You may have a rather wide nose in the case of empyema, but you do not have that dry, trophic look that you have in ozæna. The only other nasal discharge that has an offensive smell is that of syphilis, and that you can often tell by necrosed bone in the septum itself. If there is ulceration of the turbinate, you can always suspect syphilis with a good deal of probability. Of course, I am simply referring to the antrum; the other cavities have to be considered in your examination. The ethmoid will also produce a bad-smelling pus, but, in my experience, not nearly so bad as the antrum.

*Dr. Taft.*—One feature of the diagnosis would be that in empyema the odor of the discharge is evident to the patient; in chronic nasal catarrh the odor is not perceptible to the patient.

*Dr. Brackett.*—I move a cordial expression of thanks to the gentleman who has read the paper, and given us these most interesting demonstrations and diagnoses.

Unanimously carried.

William H. Potter, D.M.D.,
*Editor American Academy Dental Science.*

